# A General Cross-Layer Cloud Scheduling Framework for Multiple IoT Computer Tasks

**DOI:** 10.3390/s18061671

**Published:** 2018-05-23

**Authors:** Guanlin Wu, Weidong Bao, Xiaomin Zhu, Xiongtao Zhang

**Affiliations:** 1 College of Systems Engineering, National University of Defense Technology, Changsha 410073, China; wdbao@nudt.edu.cn (W.B.); xmzhu@nudt.edu.cn (X.Z.); zhangxiongtao14@nudt.edu.cn (X.Z.); 2 State Key Laboratory of High Performance Computing, National University of Defense Technology, Changsha 410073, China

**Keywords:** IoT services, cross-layer cloud computing, general scheduling framework, computer task, specific scheduling models and algorithms

## Abstract

The diversity of IoT services and applications brings enormous challenges to improving the performance of multiple computer tasks’ scheduling in cross-layer cloud computing systems. Unfortunately, the commonly-employed frameworks fail to adapt to the new patterns on the cross-layer cloud. To solve this issue, we design a new computer task scheduling framework for multiple IoT services in cross-layer cloud computing systems. Specifically, we first analyze the features of the cross-layer cloud and computer tasks. Then, we design the scheduling framework based on the analysis and present detailed models to illustrate the procedures of using the framework. With the proposed framework, the IoT services deployed in cross-layer cloud computing systems can dynamically select suitable algorithms and use resources more effectively to finish computer tasks with different objectives. Finally, the algorithms are given based on the framework, and extensive experiments are also given to validate its effectiveness, as well as its superiority.

## 1. Introduction

**Figure 1:**
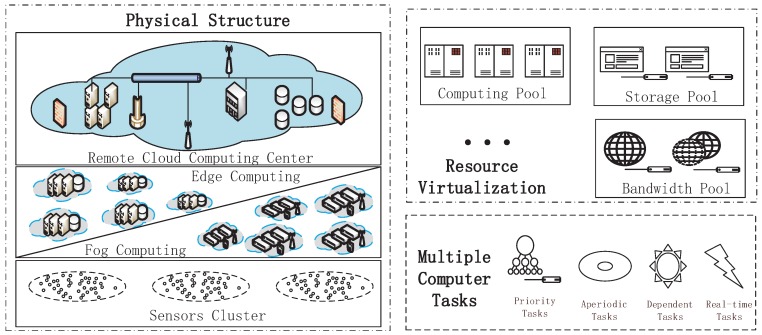
A schematic diagram of a cross-layer cloud computing system.

Recently, cross-layer cloud computing systems, as shown in Figure 1, have become more and more important in people’s daily lives, particularly with the development of various computing technologies. The cross-layer cloud computing system is a cloud computing environment that uses a mix of the local edge computing or the fog computing cloud, the remote cloud data center and other types of cloud platforms to support the device cluster. Edge computing or fog computing is usually deployed near the users and is in charge of delay-sensitive tasks and basic data pre-processing [1]. The remote cloud data center is usually responsible for computation-intensive tasks [2]. In this way, organizations are able to provide computer services with orchestration among these different platforms based on their requirements.

However, to meet the growing multiple computer demand of IoT services, the diversity of these multiple computer tasks has become an important feature of the cross-layer cloud computing system, and it makes achieving great performance more complex. On the one hand, these multiple computer tasks in the cross-layer cloud computing system differ not only in task type, but also in task scheduling objective. For instance, a dependent task is one of the most common types of computer tasks in IoT services. It can be decomposed into different tasks based on logical sequences in the calculation process, and researchers can obtain these important conclusions faster [3]. On the contrary, there is also a type of task called an independent task. Besides, long-running tasks in IoT services need fault-tolerant mechanisms and more time to reduce the expense of high rollback operations when failures occur, but real-time deadline tasks that are less sensitive to delay need less time [4]. On the other hand, different tasks in IoT services may also have common performance optimization goals, such as energy saving and system reliability. Sometimes, different tasks will select some common scheduling objectives according to their actual demands [5,6]. This makes the required algorithms comparable, and we must take this into account.

The diversity of IoT services and applications brings enormous challenges to improving the performance of multiple computer tasks’ scheduling in cross-layer cloud computing systems. Unfortunately, the commonly-employed frameworks fail adapt to the new patterns on the cross-layer cloud. Firstly, the conventional computer task scheduling frameworks do not have the ability to self-adaptively adjust. The conventional frameworks are always deployed as a monolithic system component. In this way, it is hard to adjust the optimization goals and select the most appropriate algorithm after the parameters have been set. Secondly, it is cumbersome and fallible when adding new scheduling capability in conventional computer task scheduling frameworks for cross-layer cloud computing systems. That is because there are many computer task scheduling strategies designed with different optimization objectives that share some common functional components and rely on a similar software engineering framework for implementation. Mutual influence is a very complicated phenomenon in the conventional computer task scheduling framework. Finally, the cross-layer cloud computing system and a single cloud are different in many ways. The characteristics of the cross-layer cloud computing system would require more thought.

In this study, we propose a new computer task scheduling framework for multiple IoT services in the cross-layer cloud computing system with the theory of the software engineering framework to deal with the aforementioned challenges. We first analyze the features of the cross-layer cloud and multiple computer tasks. Then, we design the scheduling framework based on the analysis and present detailed models to illustrate the procedures of using the framework. With the proposed framework, the IoT services deployed in cross-layer cloud computing systems can dynamically select suitable algorithms and use resources more effectively to finish computer tasks with different objectives. Finally, the algorithms are given based on the framework and the typical examples of different computer tasks. Extensive experiments are also given to validate its effectiveness, as well as its superiority. The main contributions of the study are as follows:

- We analyze the features of the cross-layer cloud and computer tasks. The theoretical analysis illustrates that these components influence the resource management in the cross-layer cloud computing system directly, and they are the most basic contents of the computer task scheduling framework in the cross-layer cloud computing system.
- We design a general computer task scheduling framework for the cross-layer cloud computing system. With the scheduling framework, the computer tasks in cross-layer cloud computing systems can dynamically select suitable algorithms and use resources more effectively to solve these tasks with different restrictions of the objectives.
- We present specific models and examples to illustrate the detailed procedures used, and the corresponding algorithms are designed as a demonstration based on these theories and models. These can prove that the framework is actually executable in cross-layer cloud computing systems, and the scheduling objectives are able to be dynamically matched to different types of tasks.
- We evaluate the framework with extensive experiments. These two examples represent different typical scenarios, and the experimental results prove its effectiveness, as well as its superiority.

The remaining part of the paper is divided into six sections. Relevant works are briefly reviewed in Section 2. The features of the cross-layer cloud and multiple computer task and the proposed framework are presented in Section 3. Section 4 presents specific scheduling models. Section 5 gives the detail algorithms that are contained in the algorithm pool. Section 6 presents the evaluation results of the experiments. Section 7 concludes our study.

## 2. Related Work

Under the background of cross-layer cloud computing, researchers have proposed many computer task scheduling approaches. The works related to the cross-layer cloud that are helpful to our study will be evaluated in this section.

With the rapid development and application of the cross-layer cloud in actual production, some scholars have carried out some research on the cloud computer task scheduling framework. Ergu et al. [7] divided tasks into different task pools and then constructed comparison matrices according to the task’s network bandwidth requirements, completion time, computational cost and task credibility. By the method, multiple computer tasks’ scheduling can meet the requirements of multi-objective tasks. However, when the number of tasks is large, the comparison matrix is larger and the solution speed is slower. Tang et al. [8] proposed and analyzed a two-sided bidding mechanism allowing each demander to bid to select a demand resource-price function and each supplier to bid to select a supply resource-price function. Zhu et al. [9] proposed a universal framework to schedule tasks and provide resources in the cloud computing systems to improve system performance. It sets a good example for our work to deal with the challenge in the cross-layer cloud. Wu et al. [10] proposed a collaborative multiple computer task scheduling architecture, in which heterogeneous information of nodes was considered and integrated to make decisions. Sun et al. [11] proposed a real-time and energy-efficient multiple computer task scheduling and optimization framework. Rao et al. [12] regarded computer task scheduling as a distributed learning task and proposed an algorithm to efficiently express learning experience as the core of the VM-side learning engine. In this mechanism, each VM or instance can make any resource request based on its own needs, and the request can be evaluated and automatically responded to. However, these methods use the unchangeable task pool to process batch tasks that are not suitable for the task that dynamically joins the situation. In addition, these frameworks also have the main drawback that the computational complexity is high and is mainly optimized for isomorphic resources. When the size of resources increases or heterogeneous resources are used, the performance drops rapidly. This is exactly what the cross-layer cloud often faces.

There are also some works that study the specific algorithms of computer task scheduling in the cloud environment. Farahabady et al. [13] explored how resources in the cross-layer-cloud environment should be used to run bag-of-tasks applications. Considering the deadline and the cost limitation, Zuo et al. [14] put forward a multi-objective scheduling method oriented by tasks based on ant colony optimization in a cross-layer cloud environment, with the purpose to optimize the restricted pool of edge and public cloud computing resources. Yuan et al. [15] proposed a temporal task scheduling algorithm that combines the energy price of edge cloud resources with the execution price of public cloud resources. Chang [16] studied the task scheduling algorithm and resource allocation algorithm to minimize data transmission energy cost in the cross-layer cloud. Zheng et al. [17] focused on how to improve the performance of social welfare, enlarge cloud bandwidth utilization, as well as increase the satisfaction ratio of the tenant in their study. The method aims to minimize the computational cost and time. An optimization model is established, which can obtain the approximate global optimal solution according to the situation of tasks. The above specific multiple computer task scheduling methods focus on how to adjust the allocation of resources according to the specific requirements of a certain service. They do not consider the universal heterogeneity of tasks in an actual cross-layer cloud system and, therefore, do not meet the diverse needs of cloud users.

Furthermore, traditional methods to solve multi-objectives include the weighting method [18,19], the restraint method [20] and the linear programming method [21]. In addition, many research works have studied intelligent optimization algorithms and proposed innovative algorithms for multi-objective optimization, including multi-objective optimization based on evolutionary algorithms [22], multi-objective optimization based on particle swarm optimization [23] and multi-objective optimization based on the ant colony algorithm [24]. Besides, considering multiple goals and satisfying the impact on task scheduling at the same time, Van et al. [25] proposed a binary scheduling algorithm based on the key factors in the computing system environment. However, when the amount of tasks or the type of resources is large, the time required by these algorithms to schedule resources is quite large. In particular, cloud-edge computing systems often have the characteristics of a large number of heterogeneous nodes, which makes these algorithms inadequate to meet the needs of the actual business. Different from previous research, the framework proposed in this paper pre-processes cloud-edge computing system resources before multi-objective optimization and combines resources’ heterogeneity characteristics to reasonably divide resources and task types. Therefore, it can efficiently find out the resource scheduling method to meet the multi-QoS requirements combined with the multi-objective requirements of users in the heterogeneous resources environment. This is of great significance for the improvement of cloud-edge computing system performance.

## 3. General Framework Design

In this section, we analyze the features of the cross-layer cloud and multiple computer tasks firstly, which includes task scheduling objectives, task types and resource characteristics. Then, based on these results, a detailed description of the framework is given.

### 3.1. Task Scheduling Objective

The task scheduling objective is set by a cross-layer computing system to meet the requirements of certain computer task types and their own goals. The cross-layer computing system, which is given more flexibility in task scheduling and resource collaboration, is a complex system with many different objectives in various scenes. Specifically, it can be divided into service level agreement, energy conservation, reliability and uncertainty.

#### 3.1.1. Service Level Agreement

IoT services issue the Service Level Agreement (SLA) which, as a human-readable document, is used to give a description of cloud-based IT resources in terms of the guarantees, restrictions and Quality-of-Service (QoS) features. SLA uses service quality metrics to express measurable QoS characteristics. The cross-layer computing systems’ mangers put it into these objectives to ensure that they are fulfilling the contractual QoS requirements that are published in SLAs.

#### 3.1.2. Energy Conservation

Generally, wholesalers will buy products in bulk if the price is low. Similarly, the cross-layer computing system provides more price options for users. For instance, as we know, both the unnecessary usage of computational resources and the computing migration between clouds and sensor clusters consume a large amount of energy [26].

#### 3.1.3. Reliability

Reliability is an important performance measure of cross-layer computing systems and an important indicator that IoT services must consider. The mission for combining cloud computing and edge computing is to provide a more efficient method for data processing. At this point, once errors have appeared, this will bring a devastating disaster to cross-layer IoT services. Therefore, providing corresponding fault tolerance mechanisms in both cloud computing and edge computing to improve system reliability is a key issue that cross-layer computing systems must address [27].

#### 3.1.4. Uncertainty

Controlling uncertainty within an acceptable range is another issue that cross-layer computing systems need to address. Studying how to measure and control the uncertainty is an indispensable method to effectively improve the performance of the cross-layer system. For example, the performance of remote cloud and edge clouds is constantly changing at runtime. If the uncertainty caused of system performance cannot be accurately measured at this time, the system’s scheduling scheme will be very difficult to implement and even have a negative effect on tasks’ allocation and operation [28].

### 3.2. Task Type

The requirements of different computer tasks are transported to the cross-layer system for processing and scheduling. In general, we divide computer tasks into four aspects to facilitate the processing and scheduling of computer tasks.

#### 3.2.1. Independent and Dependent Tasks

Dependent task requirements can be divided into different types of tasks or their combinations. Sometimes, the users’ service needs can be transformed to a single task directly, such as the calculation service of checking the energy account balance. In addition, sometimes it needs to be divided into multiple interrelated tasks to operate, e.g., waking up sensors and other devices in smart home can be decomposed into wake up and initialize different sensors or other devices successively. We define that a single task as an independent task and the others as dependent tasks (be denoted as a directed acyclic graph).

#### 3.2.2. Real-Time Tasks and Non-Real-Time Tasks

Based on the time constraint for completion, tasks can be divided into real-time tasks and non-real-time task. When their deadline is constrained to a certain time, we call them real-time tasks [29]. Conversely, when their constraint to be completed is infinity, they are called non-real-time tasks.

#### 3.2.3. Periodic and Aperiodic Tasks

Prediction of the finish time of periodic tasks is available since timeslots between any adjacent tasks are usually fixed. Therefore, once you know the initial task’s detailed information, you can calculate the finish time of others. On the contrary, the finish time of non-periodic tasks cannot be predicted. For instance, some system calibration procedures are typical periodic tasks, and the processing services for mobile payments are usually aperiodic tasks.

#### 3.2.4. Priority and Non-Priority Tasks

The priority of tasks can be given by the cross-layer computing system or the users directly. It comes from the agreement between services and the cross-layer computing system or can be calculated based on task characteristics in a specific system [30]. For example, the deadline of tasks can be the reason for giving priority. As for the non-priority tasks, they only need to wait to be executed in normal process queues.

### 3.3. Resource Characteristics

In the cross-layer computing system, there are two main features that should be considered in the resource scheduling process, and their differences between the local edge cloud and remoter cloud data center should be compared. We use virtualization to address those resources that process tasks in different clouds and dynamic resource provisioning to demonstrate the resource’s characteristics while provisioning by different clouds.

#### 3.3.1. Virtualization

Virtualization is the foundation of the cross-layer system to provide resilient computing services and capabilities. Although the virtualization degree of the remote cloud data center is usually higher than the local edge cloud, we can regard all of them as one big virtualized resource pool. When providing computing services, multiple Virtual Machines (VMs) or containers are established directly at hosts in the local edge cloud or remote cloud data center. Then, they are used as the basic instances of resources to handle tasks rather than physical hosts.

#### 3.3.2. Dynamic Resource Provisioning

The edge cloud can use resources from remote clouds through task migration. In this way, through migrating tasks from edge clouds to the remote data center or vice versa, resources supported by the cross-layer computing system can be dynamic and scalable based on the sensor’s demand and the cloud’s status. Dynamic resource provisioning is one of the main features of the cross-layer system, which is a significant advantage compared with conventional patterns [31].

### 3.4. Scheduling Framework

**Figure 2:**
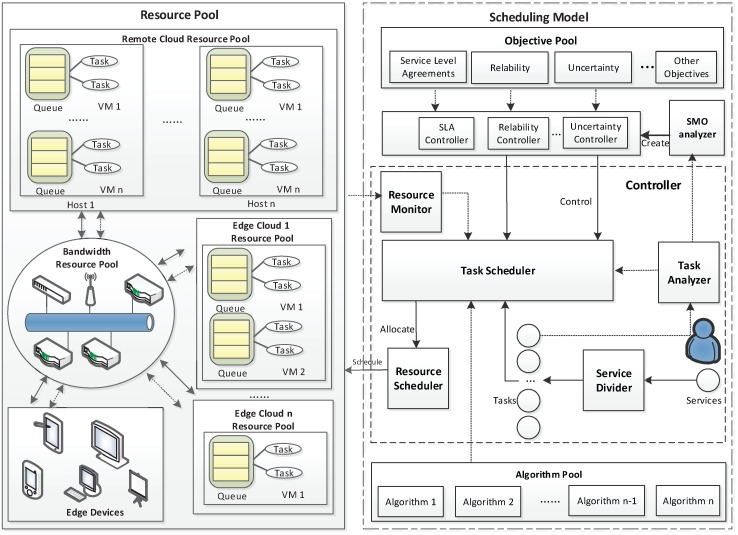
The overview of the scheduling framework.

Based on the results of the feature analysis, we design a new multiple computer task scheduling framework for cross-layer cloud computing systems, and it has the ability to be dynamically customizable. Figure 2 shows the overview of the scheduling framework, and it works with the following steps:

Step 1: Demands of different IoT services and applications are decomposed into different computer task types, and a snapshot is created by the service divider, in which the relationship between tasks is described by a directed graph.

Step 2: The task analyzer analyzes the features of computer tasks and attributes various labels to them, including task scheduling objectives, task type, and so forth.

Step 3: The task analyzer distributes the snapshot of tasks to the Scheduling Management Objectives (SMO) analyzer and task scheduler. Besides, tasks are also distributed to the task scheduler.

Step 4: The SMO analyzer can tell the differences between the objectives of each task. The SMO analyzer is an important component that we design for our proposed scheduling framework. The SMO analyzer is responsible for deciding which objectives should be selected. The SMO decision can be derived from three aspects, i.e., task nature, resource state and system hierarchy. The SMO analyzer creates a corresponding objective controller form. The objective controller selects objectives from the objective pool based on the objective controller form.

Step 5: Thisobjective controller monitors the task scheduler to make sure the output of each task is acceptable.

Step 6: The task scheduler performs arithmetic functions with the algorithms from the algorithm pool. Then, it sends information from the resource monitor and the related algorithms’ results to the resource scheduler.

Step 7: The resource scheduler allocates resources to these tasks based on the algorithms’ results from the task scheduler.

It is also worth noting that the purpose of the task scheduler is to decide how much of the resources should be allocated to the task, and the resource scheduler schedules resources to perform tasks according to the requests from the task scheduler.

## 4. Specific Examples and Models

To demonstrate our framework in detail, we use our framework to allocate the computing resource dynamically based on specific tasks. We use Example 1 as the instance of processing an independent task, and it focuses on controlling the uncertainty. Example 2 is for dependent tasks with the aim of good system reliability.

We use ζ as the component in the cross-layer cloud computing system. It can be the remote cloud $C_{0}$, a certain edge cloud from the edge cloud set $\left\{C_{1},\ldots ,C_{n}\right\}$ or an end sensor or other device from the end sensors and other devices set $\left\{Ed_{1},\ldots ,Ed_{n}\right\}$. Formally, we have ζ∈N, $N=\{C_{0},C_{1},\ldots ,C_{n},Ed_{1},\ldots ,Ed_{n}\}$, and consider that the resources in the cross-layer cloud computing system are CS(). Assume that both the remote and edge cloud in the cross-layer cloud computing system have many hosts to provide computing resources, which can be divided into many VMs or containers. Therefore, a given cloud can be regarded as a set of hosts $\left\{H_{1},H_{2},\ldots ,H_{n-1},H_{n}\right\}$, and hosts’ computing resource can be regarded as the set of VMs $\left\{VM_{1},VM_{2},\ldots ,VM_{n-1},VM_{n}\right\}$. The relationship of the computing resources between the cloud, host and VM can be denoted as follows:

(1) $$\begin{array}{c} CS\left(\zeta ,H_{1}\right)=\sum_{i=1}^{m}CS(\zeta ,H_{1},VM_{i}),\\ CS\left(\zeta \right)=\sum_{i=1}^{n}CS(\zeta ,H_{i}). \end{array}$$

### 4.1. Example 1

Example 1 takes the uncertainty as an important objective. It considers that the time-varying of CPU performance $cp_{ij}$ in VM ${VM}_{ij}$ is the reason for the uncertainty in the resource pool. Besides, the size of a specific task ${ta}_{k}$ cannot be measured accurately. Furthermore, the start time, execution time and finish time for tasks in the waiting queue are various, as well. Thus, the uncertainty from tasks should be calculated. We denote ${\tilde{cp}}_{ij}$ as the uncertain performance. Similarly, $\tilde{size}\left({ta}_{k}\right)$ represents the uncertain task size; $\tilde{st}\left({VM}_{ij},{ta}_{k}\right)$ represents the uncertain start time of task ${ta}_{k}$ in ${VM}_{ij}$; $\tilde{et}\left({VM}_{ij},{ta}_{k}\right)$ is marked as the uncertain execution time of task ${ta}_{k}$ in ${VM}_{ij}$; $\tilde{ft}\left({VM}_{ij},{ta}_{k}\right)$ is marked as the uncertain finish time of task ${ta}_{k}$ in ${VM}_{ij}$. Therefore, we have:

(2) $$\begin{array}{c} cp_{ij}=\left[c{p_{ij}}^{-},c{p_{ij}}^{+}\right],\\ size\left(ta_{k}\right)=\left[size{\left(ta_{k}\right)}^{-},size{\left(ta_{k}\right)}^{+}\right], \end{array}$$

where $size{\left({ta}_{k}\right)}^{-},size{\left({ta}_{k}\right)}^{+},cp_{ij}^{-},cp_{ij}^{+}>0$. The uncertain execution time $\tilde{et}\left({VM}_{ij},{ta}_{k}\right)$ is:

(3) $$\begin{array}{c} \tilde{et}\left({VM}_{ij},{ta}_{k}\right)=\left[size{\left({ta}_{k}\right)}^{-}/{{cp}_{ij}}^{-},size{\left({ta}_{k}\right)}^{+}/{{cp}_{ij}}^{+}\right] \end{array}$$

Therefore, the uncertain finish time $\tilde{ft}\left({VM}_{ij},{ta}_{k}\right)$ is:

(4) $$\tilde{ft}\left({VM}_{ij},{ta}_{k}\right)=\tilde{st}\left({VM}_{ij},{ta}_{k}\right)⊕\tilde{et}\left({VM}_{ij},{ta}_{k}\right).$$

In terms of a real-time task scheduling, if the task $ta_{k}$ is completed at or earlier than the deadline, task $ta_{k}$ will be finished successfully. The label of task $z({VM}_{ij},{ta}_{k})$ can be determined by the following principle:

(5) $$\left\{\begin{array}{cc} 1, & \mathrm{if}(\left(ft\left({VM}_{ij},{ta}_{k}\right)\leq d_{k}\right)\\ & \mathrm{and}\;({ta}_{status}{\left(t\right)}_{k}=1)),\\ 0, & \mathrm{otherwise}. \end{array}\right.$$

Thus, the quantity of successfully-finished tasks is to be maximized:

(6) $$\begin{array}{c} \max\sum_{k=1}^{\left|TA\right|}\sum_{i=1}^{|H_{a}|}\sum_{j=1}^{|VM_{i}|}\frac{z(VM_{ij},ta_{k})}{\left|TA\right|}|\sum_{i=1}^{|H_{a}|}\sum_{j=1}^{|VM_{i}|},\\ s.t.:z\left(VM_{ij},ta_{k}\right)\leq 1,\forall ta_{i}\in TA. \end{array}$$

Then, we consider the energy conservation as another task scheduling objective; the sum of energy consumption in the system should be minimized while scheduling:

(7) $$\min\sum_{i=1}^{|H_{a}|}\int_{st}^{ft}\left(\alpha \cdot pow_{i}\cdot x_{i}^{t}+\left(1-\alpha \right)\cdot pow_{i}\cdot u\left(t\right)\right)dt,$$

where $x_{i}^{t}\in \left\{1,0\right\}$ is the “power on” status of host $h_{i}$ in time slot *t*. α is the rate of energy consumption in an idle host. $pow_{j}$ means the overall power consumption of a host. u(t) represents host $h_{i}$’s CPU utilization at time *t*.

### 4.2. Example 2

In this example, we create a task scheduling objective based on reliability. As there is a small chance that two hosts can fail at the same time, it is assumed that when the primary copies are in the host that fails, backup copies will achieve successful completion. We consider the reliability when a host fails at once, which can be ensured by a Primary/Backup (PB) model. By using the PB model, any task ${ta}_{k}$ will have a corresponding backup ${ta}_{k}^{B}$. If ${ta}_{k}$ has encountered an accident before it is finished, ${ta}_{k}^{B}$ will be wakened. To distinguish parameters between primary and backup, we us ${\left(\right)}^{B}$ to represent backup as ${ta}_{k}^{B}$.

The backup task ${ta}_{k}^{B}$ can be executed either actively or passively. Its execution mode $mode({ta}_{i}^{B})$ is determined by the following principle:

(8) $$mode\left({ta}_{i}^{B}\right)=\left\{\begin{array}{cc} passive & \mathrm{if}\;d_{k}-ft_{k}\geq et{\left({VM}_{ij},{ta}_{k}\right)}^{B},\\ active & \mathrm{otherwise}. \end{array}\right.$$

where $ft_{k}$ represents the predicted ${ta}_{k}$ end time. Therefore, the actual execution time $et^{a}{\left({VM}_{ij},{ta}_{k}\right)}^{B}$ of ${ta}_{k}^{B}$ is:

(9) $$\begin{array}{c} \left\{\begin{array}{cc} et{\left(VM_{ij},ta_{k}\right)}^{B} & z(VM_{\mathrm{ij}},ta_{k})=0.\\ \left(0,et{\left(VM_{ij},ta_{k}\right)}^{B}\right] & z(VM_{\mathrm{ij}},ta_{k})=1\\ & \mathrm{and}\mathrm{mode}({\mathrm{ta}}_{i}^{B})=\mathrm{active}.\\ 0 & \begin{array}{c} z(VM_{\mathrm{ij}},ta_{k})=1\\ \mathrm{and}\mathrm{mode}({\mathrm{ta}}_{i}^{B})=\mathrm{passive}. \end{array} \end{array}\right. \end{array}$$

The sum of the actual execution time of the backup tasks is to be minimized. Thus, the objective is:

(10) $$\min\sum_{k=1}^{\left|TA\right|}\sum_{i=1}^{|H_{a}|}\sum_{j=1}^{|VM_{i}|}et{\left({VM}_{ij},{ta}_{k}\right)}^{B}\cdot ac_{ij}^{B}$$

where $ac_{ij}=1$ when $ta_{i}$ is allocated to resource $\zeta_{j}$ at time *t*.

## 5. Algorithm Design

We discuss three task scheduling algorithms, EASU (Energy-Aware Scheduling under Uncertainty), RAS (Reliability-Aware Scheduling) and the basic resource provisioning algorithm in this section. The EASU and RAS algorithms correspond to Example 1 and Example 2 and can be easily added into the algorithm pool in the architecture we setup.

### 5.1. EASU Algorithm

To process the uncertainty in the system, WQ, a waiting queue, and UQ, an urgent queue, are defined. When the tasks’ deadline is urgent in WQ, they will be sent to UQ. We define that a task is urgent when its laxity $L_{k}$[32] reaches the given threshold $L_{d}$. The threshold is the time cost to set up a VM on a shutdown host. We have the EASU pseudocode as follows:

In this Algorithm 1, the uncertainty is measured by the interval number. $ft_{ijk}^{-}$ and $ft_{ijk}^{+}$ represent the minima and maxima of the estimated finish time. By checking all VMs’ $ft_{ijk}^{-}$ and $ft_{ijk}^{+}$ (as shown in Lines 4–9), $ft_{min}$ and $ft_{max}$ are recorded. If $ta_{k}$’s deadline $d_{k}$ is larger than or equal to $ft_{max}$ and is shorter than or equivalent to $d_{k}$, then the task will be scheduled for a VM that has minimal energy consumption (as shown in Lines 10–11). If $d_{k}$ is between $ft_{max}$ and $ft_{min}$, EASU will choose a VM holding the minimal $ft_{ijk}^{+}$ (as shown in Lines 12–13). Otherwise, the task $ta_{k}$ will be rejected, while $L_{i}>L_{d}$ means that it is feasible to firstly launch a host and accordingly create a new VM to operate $ta_{k}$ (as shown in Lines 15–18). In the resource provisioning algorithm, the function scaleUpResources() will be introduced.

**Algorithm 1:** Pseudocode of EASU.

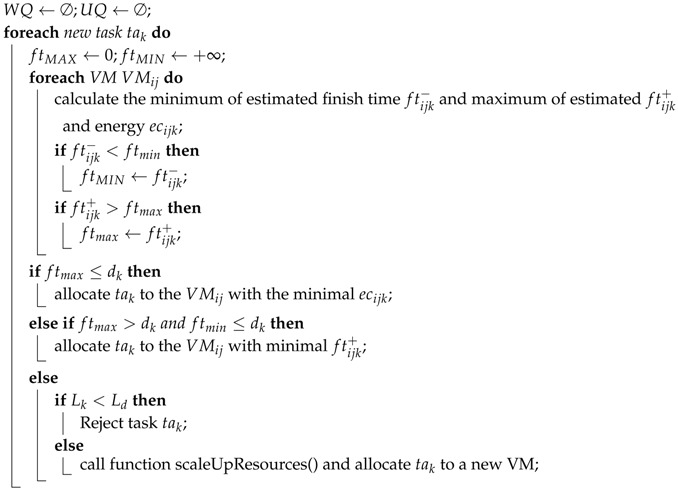



### 5.2. RAS Algorithm

Example 2 involves using the PB model to ensure the reliability-aware scheduling, and the primary tasks and backup tasks need to be respectively scheduled. Algorithm 2 shows the pseudocode of primary scheduling in RAS.

In this algorithm, the top α% hosts holding less primaries are selected as candidate hosts (as shown in Line 1), firstly. Then, we choose the VM that can complete primary tasks at the earliest time (as shown in Lines 4–11). If candidate hosts have no VM to complete primary tasks prior to the deadline, the next top α% hosts will be selected for the convenience of the next round of the search (as shown in Lines 12–15). In this way, the primary tasks can achieve an even distribution. In the case of no existing VMs able to accommodate any primary task, the scaleUpResources() function will be called (as shown in Line 17).

We omitted the backup scheduling algorithm similar to Algorithm 2. It is worth noting that it is impossible for a backup to schedule on a host that sees the allocation of primary task. In addition, a certain number of dependent constraints should be integrated into the backup scheduling algorithm.

**Algorithm 2:** Pseudocode of primaries scheduling in RAS.

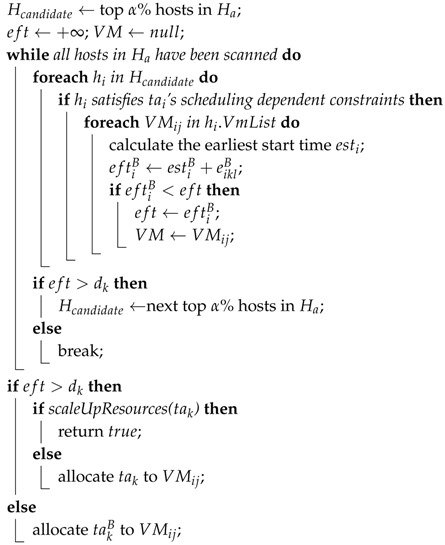



### 5.3. Resource Provisioning Algorithm

There are two functions in the resource provisioning algorithm, scaleUpResources() and scaleDownResources(), which are proposed based on the prior algorithms.

As shown in Algorithm 3, function scaleUpResources() can select a host with the highest probability of holding the lowest accommodating ability to $VM_{j}$ (as shown in Lines 3–5). With no such host being observed, it will migrate the VM (see Lines 7–11). Then, it will be checked if this migrated VM $VM_{j}$ is able to be added to the host. Thus, the $VM_{j}$ will be created to check if the task is able to achieve completion on $VM_{j}$ prior to the deadline or not (as shown in Lines 12–15). When the migration is infeasible or tasks are unable to achieve successful completion, h host $h_{i}$ will be launched, and a $VM_{ij}$ will be created. Subsequently, it is necessary to check if tasks can achieve successful completion on $VM_{ij}$ (as shown in Lines 16–20).

As for the scaleDownResources() function shown in Algorithm 4, the VM will be deleted if its ideal time exceeds the threshold (as shown in Lines 2–4). Host with no VMs will be shut down (as shown in Lines 5–7). In the case that all VMs on a host in SH are able to be migrated to one or more hosts in DH, these VMs will be migrated to a destination host, and the outset host will be closed after the migration. In contrast, all VMs will fail in migrating when a certain number of VMs is unable to migrate from the outset host (as shown in Lines 10–21). 

**Algorithm 3:** Pseudocode of scaleUpResources().

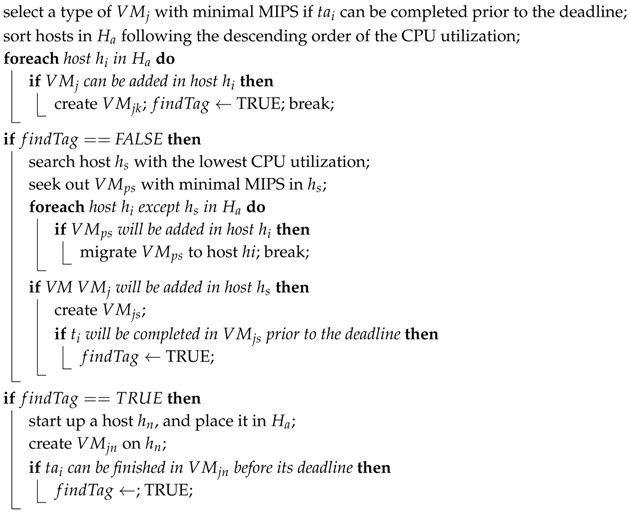



**Algorithm 4:** Pseudocode of scaleDownResources().

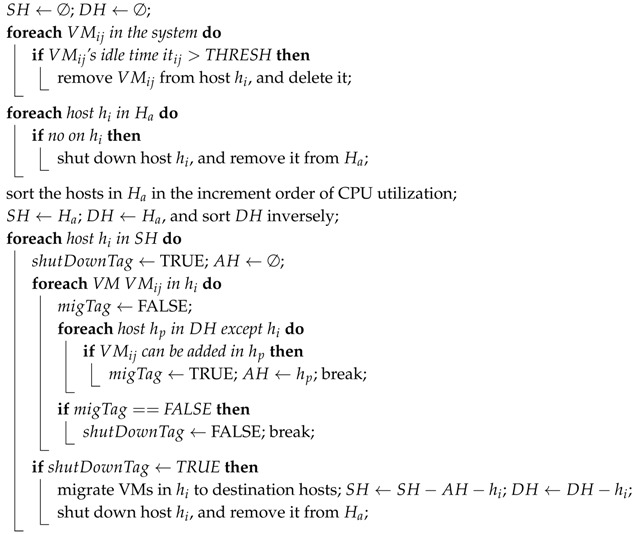



## 6. Framework Implementation

In this section, we conduct our simulation experiments with the simulation platform CloudSim toolkit and set the parameters of the cloud environment on the basis of Apache CloudStack 4.2.0. CloudStack is a kind of open source software designed to deploy and manage large networks of virtual machines, as a highly available, highly scalable Infrastructure as a Service (IaaS) cloud computing platform. CloudStack has been widely deployed in IoT environment to provide multiple cloud services. By setting the cloud environment parameters based on CloudStack, it can make our method be more easily deployed in a real system.

The physical host in CloudSim was equipped with 500 G of disk storage, 3.7 G of memory and a i3, 3.9-GHz quad-core CPU. Its peak power is 200 W. Each VM has two 23.9-GHz CPU cores and 1.5 G of memory. According to [32], the machines operated with a 1-Gbps Ethernet network. CloudSim is an extensible simulation toolkit that supports both system and behavior modeling of different cloud system components [33]. It implements generic application provisioning techniques and can be extended with ease and limited effort. We can easily simulate different clouds in the IoT environment based on CloudSim. With the simulation platform CloudSim, we set the “cloudletList()” component to simulate the workload collected from the sensor clusters. Combining the “CloudSim.clock” and “CloudSim.processEvent()” components, the operating properties of our method under different conditions can be sufficiently tested with the simulation environment.

In addition, we set different types of workloads for different test targets. For Example 1, the arrival rate is determined with the Poisson distribution, and the average amount of the task counts is set as 5000. Considering the features of the realistic workload, intervalTime determines the time interval between two sequential tasks, and intervalTime = 0.5 s in the study. For Example 2, the workload is represented by the DAG count, and we assume each task set T(or a DAG job) has random precedence constraints that are generated by the steps in [34]. The DAG size is determined by the message count *M*, and we have M=θ×N, where θ represents the degree of task dependence and *N* is set as 200. In the experiments, we test the performance impacts of the workload by setting different DAG counts and values of θ.

### 6.1. Parameter Setting and Experimental Results of Example 1

Since Example 1 takes the uncertainty as an important objective, we denote vmUncertainty as the upper bounds of the uncertainty of VMs in the system. The lower bound and upper bound of the performance of a VM are shown as follows:

(11) $$cp_{ij}^{-}=cp_{ij}^{+}\times \left(1-U\left[0,vmUncertainty\right]\right).$$

where U[0,vmUncertainty] represents an evenly-distributed random variable between zero and vmUncertainty and $cp_{ij}^{+}$ represents the required capacity for $VM_{ij}$ of the CPU.

Based on the real-time nature of the tasks, we define the deadlineBase for controlling the deadline of a task. It is used to check that the task has a loose or tight deadline:

(12) $$d_{i}=a_{i}+U\left[deadlineBase,a\times deadlineBase\right].$$

where deadlineBase is set at 400 s and *a* is set at four. The arrival rate is determined with the Poisson distribution. The intervalTime determines the time interval between two sequential tasks, and intervalTime = 0.5 s in the study.

The EASU is compared to the algorithm NMEASU and the Earliest Deadline First algorithm (EDF). NMEASU is a scheduling algorithm that schedules tasks to virtual machines with the earliest starting time, which we developed based on the OUD-OLBmethod in [35]. The algorithm does not use virtual machine online migration technology to merge virtual machine resources. In addition, all tasks to be executed by the algorithm are on the virtual machine. We discuss their following three metrics:

a Guarantee Ratio (GR): This refers to the ratio of tasks completed prior to the deadlines;
b Resource Utilization (RU): This refers to the average host utilization. We denote it as:
  (13) $$RU=\left(\sum_{k=1}^{\left|TA\right|}\sum_{i=1}^{|H_{a}|}\sum_{j=1}^{|V_{i}|}et_{ijk}\cdot z_{ijk}\right)/\left(\sum_{i=1}^{|H_{a}|}cp_{i}\cdot wt_{i}\right),$$
  where $wt_{i}$ represents the active time of host $h_{i}$ in the entire experimental process.
c Total Energy Consumption (TEC): This refers to the total energy consumption by hosts for the execution of the task set *T*.

#### 6.1.1. Performance Impact of the Task Deadline

**Figure 3:**
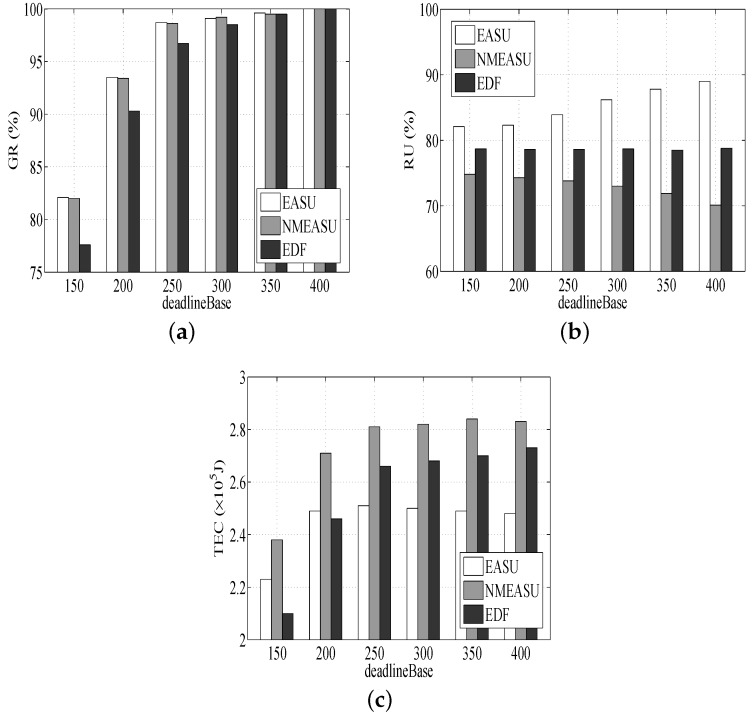
Performance impacts of task deadlines. (**a**) Impact of deadlineBase on GR, (**b**) Impact of deadlineBase on RU, (**c**) Impact of deadlineBase on TEC.

As shown in Figure 3a, all three algorithms’ GR climb as deadlineBase rises, as more tasks will be completed prior to deadlines, and the mechanism of resource scale-up is not needed. It also can be found that the GR of EASU and NMEASU is higher than EDF. The reasons can be easily found. Firstly, more tasks satisfy their deadline because EASU and NMEASU have higher priority. Secondly, EASU and NMEASU are more flexible and are able to achieve the dynamic allocation of resources to accommodate more tasks. Finally, EASU takes the uncertainties into consideration, and NMEASU has a better quality of scheduling.

According to Figure 3b, RU of three algorithms grows as deadlineBase rises. It is helpful due to the fact that the active hosts’ utilization will be higher and more tasks will be completed inside currently active hosts with no need to start more hosts, leading to looser deadlines. Similarly, since VM migration is beneficial for strengthening resources in an effective way, resources can achieve effective utilization, and the RU of EASU is higher than NMEASU. In addition, no scale-down function is deployed for EDF, causing the minimal RU.

According to Figure 3c, the TEC numbers of EASU, NMEASU and EDF increase as deadlineBase increases. The reason is that with the rise of deadlineBase, more tasks require consuming more energy. EASU consumes less energy compared to NMEASU since NMEASU is inefficient in resource utilization. EDF requires more energy than EASU once deadlineBase exceeds 200 s because it ignores energy conservation.

#### 6.1.2. Performance Impact of Uncertainty

**Figure 4:**
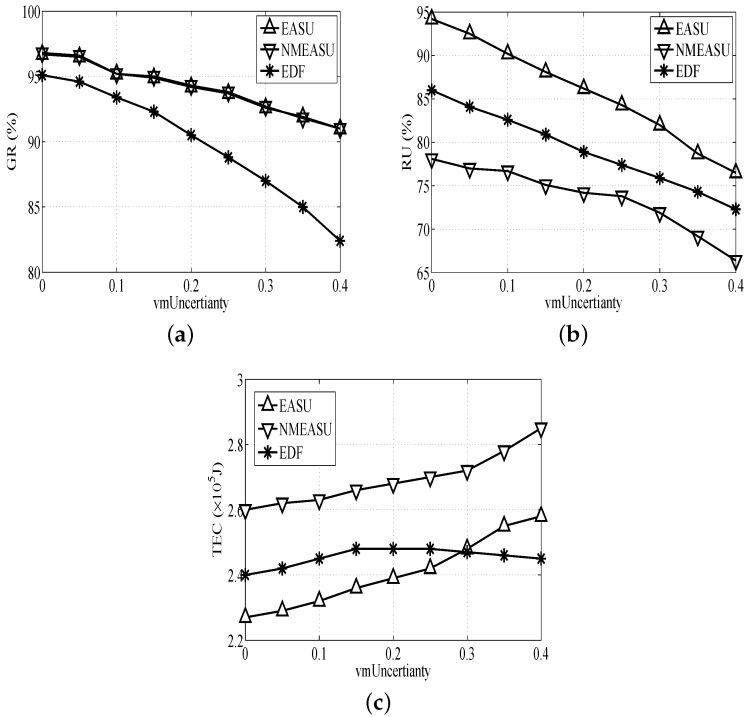
Performance impacts of task deadlines. (**a**) Impact of VMUncertainty on GR, (**b**) Impact of VMUncertainty on RU, (**c**) Impact of VMUncertainty on TEC.

Figure 4a indicates that the GR of the three algorithms drops as VMUncertainty increases, especially the EDF’s trend. This is because EDF fails to deploy strategies to manage uncertainties, which will cause really bad scheduling quality.

Based on Figure 4b, RU of the three algorithms presents an obvious decrease as VMUncertainty increases because the performance of VMs will obviously decline and require more resources of physical hosts with the rise of VMUncertainty. EASU is better than the others for deploying the uncertainty-aware and VM migration strategies.

Based on Figure 4c, the TECs of EASU and NMEASU rise because too many tasks need to be finished urgently prior to the deadlines in VMs when VMUncertainty increases, and this consumes more resources. EASU requires more energy compared with EDF when VMUncertainty exceeds 0.3. This is because EASU tends to manage the uncertainty and process more tasks without considering the energy consumption.

### 6.2. Parameter Setting and Experimental Results of Example 2

Since Example 2 pays more attention to the dependent tasks, we denote TA or a DAG job as each task set. According to [34], we set the random precedence constraints of TA. We denote the message count *M* and have M=θ×N when the number of dependent tasks *N* is given. Other parameters of Example 2 are the same as Example 1.

The RAS with the algorithm NCRAS (NCRAS simulates a non-fault tolerant real-time scheduling algorithm that is unable to tolerate any failure. It is proposed for the purpose of comparison.) is compared with the algorithm eFRD, which is an easy approach to conduct primary, as well as backup scheduling [34]. We discuss their following three metrics.

(1) Guarantee Ratio (GR): This refers to the ratio of tasks completed prior to their deadlines;
(2) Host Active Time (HAT): This refers to the total running time of all hosts in the cross-layer cloud computing system;
(3) Ratio of Task time and Host time (RTH): This refers to the rate of the execution time of all of the tasks with respect to the total active time of hosts.

#### 6.2.1. Performance Impact of the DAG Count

**Figure 5:**
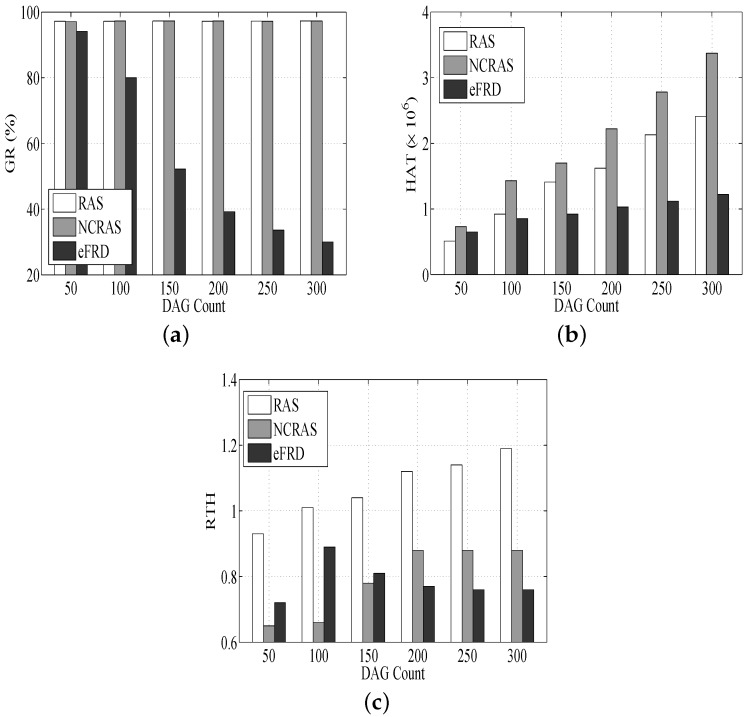
Performance impacts of the DAG count. (**a**) Impact of the DAG count on GR, (**b**) Impact of the DAG count on HAT, (**c**) Impact of the DAG count on RTH.

As shown in Figure 5a, RAS and NCRAS are basically unchangeable GRs for various DAG counts. This is due to RAS and NCRAS taking into account the limitless resources in the cross-layer cloud computing system. The increase in DAG count will make new hosts dynamically attached to more DAGs. Because eFRD cannot dynamically manage resources, the GR of eFRD drops with the rise of the DAG count.

Figure 5b illustrates that the HAT of RAS is lower than NCRAS, and it proves that RAS does better compared to NCRAS in the resource management. Furthermore, without a consolidation mechanism in NCRAS, the highest resource utilization is caused by the idle state of some resources.

In Figure 5c, the RTHs of RAS and NCRAS ascend. The RTH of eFRD rises first and then drops. This is because it is assumed that its resources are fixed in eFRD. There are enough resources to accommodate most DAGs (ranging from 50–100). However, a further rise of the DAG count, DAG count > 100, leads to the saturation of the system, long running times of hosts and a low level of resource utilization. It also can be observed that the highest RTH is in RAS, since it considers the consolidated resource mechanism.

#### 6.2.2. Performance Impact of Task Dependence

**Figure 6:**
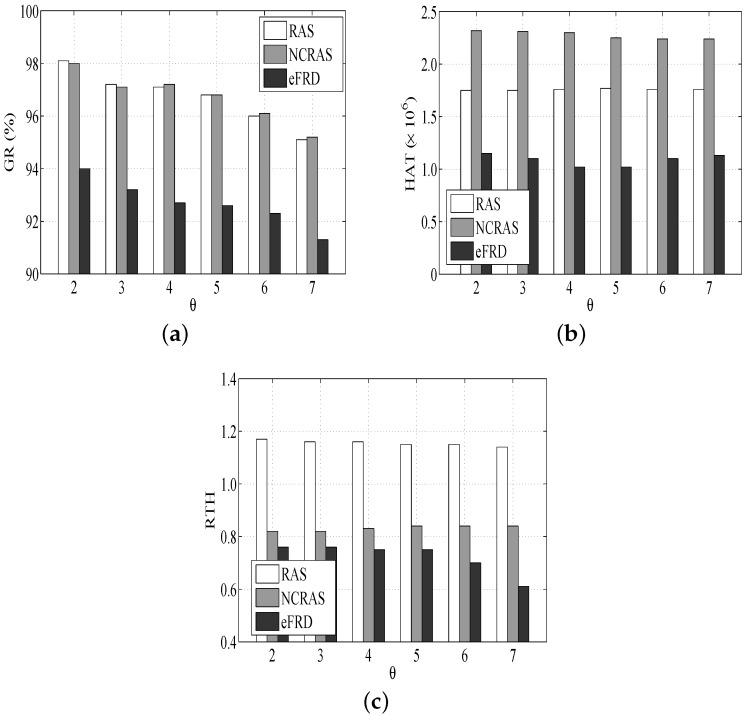
Performance impacts of Task Dependence. (**a**) Impact of Task Dependence on GR, (**b**) Impact of Task Dependence on HAT, (**c**) Impact of Task Dependence on RTH.

As shown in Figure 6a, the GR of all the algorithms modestly drops when the θ rises. The reason is that the schedulability of the system is degraded with the rise of task dependency. This is similar to the other experiments such as RAS and NCRAS.

Figure 6b illustrates that the HAT of NCRAS is the highest. Although the small task dependence is able to allow more tasks to be executed in parallel and some hosts will be released, as well as closed at an earlier time, NCRAS does not have the advantage, and it requires more resources. Hence, the difference in HAT between NCRAS and other algorithms drops with the rise of θ (this means that the number of tasks supporting parallel execution is decreased).

In Figure 6c, RAS has a higher RTH than the others, and the RTH of all the algorithms drops when θ rises. The reason is due to the high task dependence, which leads to more execution limitations and fewer tasks that are available for VMs despite their idleness. It is helpful to save resources and maintain a lower RTH, since the tasks executed in parallel drop with the rise of θ.

## 7. Conclusions

In this paper, we propose a new cross-layer cloud scheduling framework for multiple IoT computer tasks, and the IoT services deployed in cross-layer cloud computing systems can dynamically select suitable algorithms and use resources more effectively to finish computer tasks with different objectives. The features of the cross-layer cloud and IoT computer tasks are analyzed, and the scheduling framework is given based on the analysis. Then, two typical examples of different task scheduling schemes and their detailed models are proposed based on the framework so as to illustrate the procedures of using the framework. To cope with two typical examples with our framework, we proposed EASU, the RAS algorithm and the basic resource provisioning algorithm in Section 4. We compared them to the approaches previously proposed by [34,35]. The experiments’ results validate its effectiveness, as well as its superiority. Our further studies will focus on the following aspects: (1) we will combine some intelligent forecast mechanisms into our framework to make the scheduling results more precise; and (2) we will extend our framework to improve the ability of QoS estimation and management since different IoT services usually have different SLA levels.
